# The complexity of addressing equity in COVID-19-related global health governance and population health research priorities in Canada: a multilevel qualitative study

**DOI:** 10.1186/s12889-024-20893-z

**Published:** 2024-12-05

**Authors:** Muriel Mac-Seing, Erica Di Ruggiero

**Affiliations:** 1 School of Public Health & Centre de recherche en santé publique, Université de Montréal, Québec, Canada; 2 Social and Behavioural Health Sciences Division & Centre for Global Health, Dalla Lana School of Public Health, University of Toronto, Ontario, Canada; 3 Social and Behavioural Health Sciences Division, Institute of Health Policy, Management and Evaluation & Centre for Global Health, Dalla Lana School of Public Health, University of Toronto, Ontario, Canada

**Keywords:** COVID-19, Equity, Accountability, Funding, Global Health Governance, Population Health Research, Covidization, Canada, Intersectionality, Multiple streams Framework

## Abstract

**Background:**

Since COVID-19 emerged in 2020, the promotion of health equity, including in research, has further been challenged worldwide by both global health governance (GHG) processes and decisions, and national public health control measures. These global and national decisions have also led to the ‘covidization’ of health research agendas where resources have been massively channelled to address COVID-19, especially during the first years of the pandemic. This situation could potentially result in current and future population health research priorities not explicitly tackling equity as a central tenet. The study objective examined how and to what extent the COVID-19-related GHG architecture is affecting population health research priorities in Canada.

**Methods:**

We conducted a multilevel qualitative study informed by the intersectionality-based policy analysis and multiple streams frameworks. We collected and thematically analysed data from four groups of respondents (*n* = 35: researchers, research funders and global and public health research institutes in Canada, and WHO/international actors) and an interactive feedback workshop (*n* = 40 participants).

**Results:**

Study findings generated four main themes. First, both global and national COVID-19 responses failed to address equity considerations, especially among populations in situations of vulnerability and marginalisation. Second, the integrated examination of funding, equity, and accountability was judged as necessary determinants of GHG and population health research priorities in Canada. Third, contrary to common beliefs about COVID-19, the consequences were not all negative, but they were also positive and unintended, and lessons can be learned. Fourth, study respondents proposed multiple recommendations to address inequities in the complex intersection between COVID-19-related GHG and population health research in Canada.

**Conclusion:**

This study provides substantial evidence of the multilayered and complex intersection between COVID-19-related GHG and population health research priorities in Canada. Although the window of opportunity was slim according to study respondents, there was still a unique collective effort to address COVID-19-related socioeconomic and health inequities by considering the numerous recommendations proposed by the four groups of study respondents. These recommendations can directly contribute to improving knowledge of global and national population health and equity research strategies in the context of an evolving pandemic and for policy- and decision-makers to adjust and rectify the course of global and public health governance.

**Supplementary Information:**

The online version contains supplementary material available at 10.1186/s12889-024-20893-z.

## Background

As of December 2023, COVID-19 has killed more than 6,900,000 people and caused more than 700 million cases in the world [1]. Since the COVID-19 pandemic emerged almost five years ago, the promotion of health equity in general, including in research, has further been challenged worldwide by both global health governance (GHG) processes and decisions, and national public health control measures, which exacerbated pre-existing social and health inequities [2]. Global health governance is understood here as “governance arrangements needed to further agreed global health goals” such as health equity, access to medicines, or social justice [3]. In this GHG definition, the emphasis on equitable health outcomes is paramount. During the pandemic, these governance (e.g., global COVID-19 guidance from the World Health Organization) and policy (e.g., national decisions to restrict mobility) decisions generated multiple and multilayered disruptions in economic, social, health, education, and environmental systems [2, 4, 5]. No one escaped the COVID-19-related consequences of massive lockdowns, school and business closures, restrictions in movement, a surge in the response of healthcare and public health systems, and exacerbation of mental health issues [2, 6]. However, many governance and policy decisions failed to recognise people’s intersectional vulnerabilities within and across countries [7]. They affected citizens in all countries irrespective of their income level [4, 6, 8]. COVID-19 has disproportionately affected populations living and working in conditions of vulnerability or marginalisation such as frontline workers [9–11], elderly people [12–14], women [15–17], children and youth [18, 19], Black, Indigenous and People of Colour (BIPOC) [20–22], people with disabilities [23, 24], homeless people [25, 26], and migrants and refugees [27, 28].

These global and national decisions and policy measures have not only exacerbated underlying social, economic, and health inequities, but they have led to the ‘covidization’ of health research agendas where resources have been reoriented to address the COVID-19 pandemic [29]. Madhukar Pai, a Canadian global health researcher working in the field of Tuberculosis, first coined the term covidization in 2020 highlighting three main risks [29]. These include a massive influx of funding towards COVID-19 research initiatives, researchers whose primary field of expertise is not COVID-19 shifting their research priorities towards COVID-19, and unreviewed preprints and research papers reviewed at an unprecedented speed flooding the publication arena [29, 30]. In Canada, the Canadian Institutes of Health Research (CIHR) cancelled its 2020 annual spring grants competition evoking the priority to ‘help stop the spread of COVID-19” and resumed its regular fall grant competition later that year [31]. In the same period in 2020, CIHR announced a new COVID-19 Rapid Research Funding scheme which awarded the equivalent of 79.4 million USD (or 111.1 million CAD) for 140 research projects which included the contribution of other funding agencies such as the International Development Research Centre (IDRC) [32]. This was an example of funding schemes being reoriented rapidly in Canada to respond to COVID-19 and that in turn might have affected both global and national public and populational health research projects [33]. In June 2022, the CIHR Institute of Population and Public Health launched its 2022–2026 strategic plan reiterating their priority in advancing equity in research funding areas such as anti-colonialism, anti-racism, and anti-ableism, and training opportunities focused on advancing equitable health outcomes and on EDI (equity, diversity, and inclusion) [34]. In other countries, researchers examined the relationships between COVID-19 and public and population health research. They reported that beyond researching COVID-19 and its impacts, authors highlighted the importance of examining non-COVID-19 public health considerations such as socioeconomic and equity aspects and health systems research [35], the determinants of vaccine hesitancy and impacts of COVID-19 on vulnerable populations [36], and interdisciplinary research to maximise our understanding of the evolving COVID-19 pandemic [37].

Framed within the above context, we sought to investigate how and to what extent the COVID-19-related GHG architecture (how it is structured and which entities it includes) is affecting population health research priorities in Canada, notably research projects’ objectives of improving population health and health equity. According to the World Health Organization, equity is defined as “the absence of unfair, avoidable or remediable differences among groups of people” and where health equity “is achieved when everyone can attain their full potential for health and well-being” [38]. Specifically, we sought to describe the features of the COVID-19-related GHG architecture in Canada and the impacts of these features on influencing research agendas and explore the consequences of COVID-19 on research projects’ population health aspects such as determinants of health and equity, and the potential solutions to address these.

## Methods

We conducted a multilevel qualitative study of the COVID-19-related GHG architecture on population health research agendas in Canada. This study is embedded in a larger study that also involves a scoping review of the relationships between COVID-19-related GHG and population health priorities (research, policy, and practice) in G20 countries [5]. It is guided by an advisory committee composed of scholars and experts in global public health, health governance, healthy public policy, and knowledge mobilisation and exchange. Our current study involved semi-structured in-depth interviews and an interactive thematic feedback workshop.

### Theoretical frameworks

We adopted the theoretical bricolage of combining and adapting both Intersectionality-Based Policy Analysis (IBPA) and Multiple Streams frameworks for their joint theoretical strengths [39] to analyse study findings. The IBPA framework critically analyses different types of inequities and intersectional vulnerabilities experienced by individuals and groups of individuals living and working in conditions of vulnerability and/or marginalisation [40]. Intersectionality was first coined by Kimberlé Crenshaw to address the multiple concomitant prejudices faced by Black American women workers who were neither protected by anti-sexism nor anti-racism policy and legislation [41]. Kingdon’s Multiple Streams Framework (MSF) approach to agenda setting postulates that when the problem, policy, and political streams meet, “a problem is recognised, a solution is available, the political conditions are right”, and all streams meet through a window of opportunity which promotes policy implementation [42]. It has been argued that COVID-19 provides an important window of opportunity through which the MSF can enable us to better understand the determinants of health and health equity in the context of GHG [43].

### Participant selection

From February 9 to August 8, 2022, we recruited participants during successive waves of COVID-19.

We conducted virtual interviews with study participants based in seven Canadian provinces and internationally in Switzerland and Senegal. We interviewed four groups of study participants: (1) Canadian researchers involved in global and/or public/population health initiatives, (2) Canadian research institutes/centres/hubs working on global public health or related health policy issues, (3) Canadian research funders, and (4) World Health Organization (WHO)/international actors. Given our commitment to include respondents with a diversity of social identities and experiences, we conducted purposive sampling to maximise variation while considering gender, ethnicity, discipline, and geography [44]. Recruitment of participants continued until thematic saturation was reached [44]. Out of 49 invitations, 35 people participated in our study, with an overall response rate of 71% and the highest acceptance rate among Canadian research funders (90%) and the least (40%) among WHO/international actors (Table 1). The main reason given for not participating in the study was the lack of time.

**Table 1:** Response rate per group of study participants

	Canadian researchers	Canadian research institutes, centres, hubs	Canadian research funders	WHO & international actors
Invitation	23	6	10	10
Response	18	4	9	4
% of response	78	67	90	40
Total	35 respondents / 49 invitations (71% response rate)			

In total, 25 (71%) respondents identified as women, and seven (20%) self-identified as Black, People of Colour, or Indigenous/Metis. The largest share of participants comes from the two most populous Canadian provinces, Ontario (17 respondents, 48.5%) where most research funders and several universities are located followed by Québec (7 respondents, 20%) (Table 2).

**Table 2:** Participant characteristics

	Canadian researchers	Canadian research institutes, centres & hubs	Canadian research funders	WHO & international actors
**Gender (35)**				
Women (25) Men (10)	14 4	2 2	9 -	- 4
**Ethnicity (other than Caucasian) (7)**				-
Black (2) Indigenous/Metis (2) People of Colour (3)	2 2 3	- - -	- - -	- - -
**Discipline (35)**				
Global/public health (14) Specific populations (4) Epidemiology/Infectious diseases (6) Social sciences (8) Environment/Biology (3)	4 4 3 6 1	2 - 1 1 -	4 - 2 1 2	4 - - - -
**Geography (35)**				
Canada (31) *British Columbia (3)* *Alberta (1)* *Saskatchewan (1)* *Ontario (17)* *Québec (7)* *Nova Scotia (1)* *Prince Edward* *Island (1)* Switzerland (3) Senegal (1)	2 1 1 7 5 1 1 - -	- - - 3 1 - - - -	1 - - 7 1 - - - -	- - - - - - - 3 1

### Data collection

MMS conducted in-depth semi-structured interviews in both English (30/35, 86%) and French (5/35, 14%), which were then transcribed by a professional bilingual transcription specialist. For quality assurance, each transcript was reviewed by MMS against its recording. The interview guide (available in the supplementary file) was informed by the IBPA and Multiple Streams frameworks and adapted for this research. Two sets of questions were asked, descriptive questions related to both the IBPA [40] and MSF questions to identify the problem and windows of opportunity [42] when examining the relationships between COVID-19-related GHG and population health research priorities in Canada, and transformative questions related to solutions and recommendations proposed by study respondents to address identified problems [40]. To further triangulate study findings [44], we conducted a 90-minute thematic feedback workshop where we presented the preliminary study findings and sought feedback and recommendations on these results from the wider global public health community (researchers, students, practitioners, funders, governmental public health bodies, and civil society). This workshop was held on November 22, 2022, at the Canadian Conference on Global Health [45], and was attended by 40 people who participated in person and virtually.

### Data analysis

We adopted a step-wise thematic analysis [46] informed by the IBPA and MSF frameworks. First, all recordings were listened to, and transcripts were read at least twice, while preliminary thoughts and ideas were noted in a logbook. Second, following a deductive-inductive approach, an initial coding tree was developed by MMS and reviewed by EDR to identify relevant data. Third, all interview transcripts were imported in NVivo (version 12), coding was performed iteratively, and emerging themes were further identified. Fourth, to review the representativeness of themes and identify potential relationships among them, the online collaborative Mural application (https://www.mural.co/) was used to visually experiment and elicit these relationships while systematically organising relevant verbatims per codes and themes identified that would explain these relationships. Fifth, the emerging themes and relationships among themes were reviewed by the authors. Sixth, we organised and analysed interview data through intersectional and multiple streams lenses. Preliminary findings were discussed during the thematic workshop to seek conference participants’ feedback and recommendations on our study findings.

### Research ethics

This study was approved by the University of Toronto Research Ethics Board (REB-42067) in February 2022.

## Results

Our study findings generated four main themes: (1) a complex COVID-19-related-GHG architecture that failed to address equity issues, (2) the intricate interrelationships between funding, equity, and accountability as essential determinants of COVID-19-related GHG and population health research in Canada, (3) the different types of consequences related to COVID-19, and (4) the recommendations of study participants to address inequities in the complex intersection between COVID-19-related GHG and population health research in Canada.

### A complex COVID-19-related GHG architecture that failed to address equity

Before the emergence of COVID-19, the GHG architecture has been described as complex and messy; these characteristics within the GHG architecture are not new [47]. A majority of respondents further expressed how “messy” the GHG architecture was during the initial years of COVID-19. To address new challenges surfaced by COVID-19, an additional layer of COVID-19-related ‘messiness’ was noted, coupled with the involvement of well-established and more recent actors working in global public health emergency preparedness and responses, global vaccination initiatives, and funding schemes. At the forefront of COVID-19-related-GHG are WHO and its representatives and associated initiatives, such as COVAX for promoting COVID-19 vaccine equity, in addition to a multitude of actors evolving within the GHG ecosystem such as the Member States, the World Trade Organization (WTO), pharmaceutical corporations, philanthropic foundations, public-private partnerships, and civil society organisations. Compounded with this architecture persists the interrogation of accountability processes among the different actors, especially amid an evolving pandemic.

*I’ll start by saying it’s a mess. And that’s partly because we are in a global health crisis. But the other two reasons it’s messy is because first of all there are just a lot of different actors*, *not only all of the different members of the World Health Organization*, *so all the countries*, *but then we have intergovernmental organisations like the World Trade Organization*, *GAVI*, *CEPI*, *the list goes on*, *and so we have to figure out what are their relationships with one another*, *who makes decisions (…). The other thing that makes it really messy is there’s no sort of established framework of accountability for how people should respond or make decisions in the pandemic.* (Respondent 5)

Another major recurring theme discussed by study respondents was the failed national and global responses to addressing equity considerations. At the national level in Canada, the reshuffling of a messy GHG architecture due to COVID-19 had a direct impact on the type of research projects which focused on population health and health equity.

*From one day to the next*, *there is no longer anything that is important. There are no longer any research priorities that make sense apart from COVID-19 (…). There are many issues that people need to deal with and be concerned about*, *such as the issue of inequality*, *maternal health*, *community health (…). The researchers wanted to focus on what allowed them to be funded. I consider this as a perverse effect.* (Respondent 34)

At the global level, these equity considerations included global COVID-19 vaccine allocation and distribution and public health measures across different regions and countries of the world or within national jurisdictions for various population sub-groups living and working in conditions of vulnerability and/or marginalisation.

*The same populations that tend to bear a greater burden of just about everything have tended to bear the greater burden of COVID and its sequelae*, *and the policies… So early on in the pandemic and throughout it*, *people who with extreme and extraordinary wealth*, *for example*, *have been able to circumnavigate the greatest harms of the pandemic restrictions.* (Respondent 14)

Moreover, global actors, state leaders, and WHO were considered incapable of supporting the Trade-Related Aspects of Intellectual Property Rights (TRIPS) waiver which could have otherwise facilitated a more equitable COVID-19 vaccine allocation throughout regions of the globe and ensured more equitable health outcomes among population groups within and across countries. Governments such as Canada opted to serve first their own citizens instead of promoting simultaneously the health of populations globally.

*We became protectionist*, *we became inward-looking as many countries did*, *many regions did*, *but we failed to support the TRIPS waiver. We kept sort of saying “Oh*, *we are not opposed to it*, *but we just don’t support it”… In not supporting it*, *you are de facto opposing it and coming up with all kinds of lame excuses.* (Respondent 15)

Underlying these conversations on equity issues also lays the omnipresence of societal power dynamics and structures (e.g., racism, sexism, ableism, and colonialism) that permeate the GHG and national public and population health architectures and how populations stay healthy or develop negative health outcomes, both in the context of COVID-19 and without COVID-19.

*So in Ontario [the largest populated Canadian province]*, *we think a lot about equity in terms of how you distribute vaccines and are they reaching the right groups (…). Do people have the ability to take time off work to go get a test? Or to stay at home if they are sick? (…) We tend to think about access to healthcare services as being an equity issue*, *less so addressing pernicious forces like racism and colonialism.* (Respondent 5)

### The intricate interrelationships between funding, equity, and accountability as essential determinants of COVID-19-related GHG and population health research in Canada

For many respondents, equity cuts across COVID-19-related GHG and population health research priorities in Canada. Equity was considered to be intertwined within a complex GHG architecture of intersectoral collaboration and coordination, partnerships, and formal and informal processes (e.g., in meetings and funding review panels). In the context of COVID-19, equity considerations of all types (gender, health, racial, socioeconomic, and others) are tied to the decisions and arrangements made by actors in global and population health, including research. These decisions impact on how Canadian researchers, research funders, and global public health research institutes and entities realise their work, that in turn affects how Canadian citizens benefit from the outcomes of population health-based research initiatives. Specifically, according to study respondents, COVID-19 has shifted research priorities in all fields including population health. Covidization not only generated an influx of funding toward COVID-19 and infectious disease research initiatives, but it seemed to have also shifted the attention of researchers and research at the expense of research on health promotion or non-communicable disease prevention, for example.

*I think it definitely shifted our understanding of and focus on what should be the first priority and I feel this is going to continue that infectious diseases will have much more of a research priority. In Western societies*, *non-communicable diseases have had a much stronger research focus*, *but maybe this could be challenged by COVID-19*, *that will be interesting to see going forward*, *how much of an impact it had in terms of the longer-term balance between the different relevant sectors.* (Respondent 1)

Equity was further described as an ethical principle whereby solidarity at both global and local levels is essential for COVID-19 vaccine access, access to therapeutics and diagnostics, and concern and care for others wherever they reside.

*[For] Any conception of equity you need a strong sense of solidarity because without solidarity nobody cares about equity. And underlying any sense of solidarity you need a strong sense of empathy. Because without empathy*, *you don’t care about your community and there is no sense of solidarity. So I think there’s a cascade of values (…). I think othering in global health is the root of a lot of problems*, *including vaccine inequity*, *colonialism*, *and racism and so to me*, *there’s a fundamental set of values there.* (Respondent 13)

In addition, study respondents were very candid about the prerequisites for equitable health outcomes to happen, that is adequate funding combined with accountability processes to analyse and assess the realisation of these outcomes, beyond good intentions and plans.

*So one of the most straightforward*, *or perhaps salient areas is just access to resources. So it’s very apparent*, *right when we allocate vaccines*, *when we allocate COVID-19 therapies*, *when we allocate personal protective equipment like masks (…)*, *right away you can sort of see inequities. You can see who is getting them*, *and who is not getting them. If we don’t have enough of these things in the first place*, *then we need to prioritise who gets them and that’s immediately where we need to start thinking about equity. But a lot of those questions don’t attend to how you fix structural things that create or exacerbate inequity.* (Respondent 5)

*We haven’t delivered on promises [regarding Canada’s contribution to COVAX]*, *the public appetite for accountability there is absent (…). There is no accountability or follow-up and there seems to be a lot of lags in memory or desire to pay attention to following this through. Why does it happen that we have all of this ineffective governance?* (Respondent 14)

Despite these challenges, several study participants agreed, at the time the interview was conducted, that COVID-19 provided a historic window of opportunity to address GHG challenges and social injustices and inequities as highlighted by the number and population groups affected worldwide. Recentering population health research priorities became essential given the salience of its key role in addressing and providing evidence and strategic orientations for improving population health amid a global health crisis. Policy- and decision-makers were avid of evidence-focused information like never before to quickly respond to the emerging planetary threat of COVID-19 and protect their economy, citizens, and political reputation. One respondent shared the unexpectedly high demand for evidence by policy- and decision-makers so they could make informed decisions.

*I think that the last two years have been like a golden age in terms of the political and policy demand for evidence (…). I have never before seen such demand for evidence and not just from the usual people but from top political leaders (…). So many political leaders have had experience and see the value of evidence and so that is why in part we feel like 2022 is the window of opportunity.* (Respondent 24).

However, the window of opportunity for an uptick in interest in evidence-informed policy-making was considered surprisingly narrow despite obvious health inequities engendered by COVID-19 throughout the first waves of the pandemic (‘Problem’), momentaneous changes in policy to fund more interventions and research that address the COVID-19 pandemic (‘Policy’), and a heightened appetite from politicians to be part of the solutions (‘Politics’). According to some respondents, the window of opportunity might be already closing, or closed. Policy- and decision-makers have scaled back many public health policy measures. Furthermore, the attention on equity issues has faded, and the population has been asking to return to ‘business as usual’, as illustrated by decision-makers intervening less at population health level and generalised fatigue of population groups to mask, physically distance, or accept to be restricted in their activities.

*COVID itself*, *which*, *you know*, *all of us who do population health work have been very used to using any topic as a window through which we can understand issues of equity*, *but I don’t know that there has been any event in the same*, *that has drawn public attention to issues of equity as well as this pandemic has and so*, *that is a bit of a lost opportunity. And maybe there is still space to resurrect it (…)* (Respondent 14).

### Consequences of COVID-19

Contrary to common beliefs about COVID-19, the consequences were not all negative. They were also positive and unintended or unanticipated and there are lessons that can be learned from COVID-19. These consequences affected the selection of population groups living in situation of vulnerability to be studied, researchers themselves, as well as which population health research priorities as illustrated below.

#### Negative consequences on specific population groups and researchers and their work

Three main negative consequences pertained to the exacerbation of inequities among populations who were already experiencing conditions of intersectional vulnerabilities or marginalisation such as people with disabilities, in addition to equity considerations not being considered nor assessed during the early onset of the pandemic.

*We did a few research projects looking at the impact of COVID-19 on children with disabilities. The loss of access to services*, *to support. Families who depend on*, *for example*, *caregivers at home could not have access to the services. And those were essential for maintaining the family to work.* (Respondent 19)

The other two negative consequences affected the researchers themselves and the scope of studies based on how research methodologies were used.

*I think there’s absolutely been a covidization and there’s been a plethora of opportunities and funding and pressure. People are exhausted and burned out (sigh)*, *well partly because people who think systems and think interconnections then get invited to be or expected to be in a million different things. In the meantime*, *you are also dealing with the impact of the pandemic on your personal life*, *on your own family and your community.* (Respondent 4)

*It has also impacted qualitative data collection. Because we communicate in so many different ways. (…). I think recruitment was really impacted because you need that personal touch in communities rather than getting an email from a person that you have never met before (…). And then*, *for quantitative surveys*, *they had to be completely redesigned. (Respondent 35)*

#### Positive consequences on public health research focus, funding, and collaboration

Despite numerous untoward consequences, study respondents also identified several positive consequences generated by the COVID-19 pandemic. One of the main positive consequences was the rediscovery of the importance of population and public health and related research, in tackling an unprecedented global public health issue.

*I think it [COVID-19] put it [public/population health] back on the agenda. I think that population health research*, *and more precisely public health research*, *was faring badly (…). It’s important to have strong public health and public health research because that’s what’s going to allow us to reduce demand and not always increase supply.* (Respondent 23)

Through covidization, another positive aspect was the rapid policy decisions made by the Canadian government and research donors to avail new research funds for researchers to respond to COVID-19. Research donors provided substantial funding to address different population and public health research priorities both at Canadian and international levels.

*All of a sudden there were tens of millions of dollars in research funding that was about COVID-19. I mean*, *it was almost impossible not to get funding for some form of COVID-19-related [work] (…). Now how much of it kind of took a strong population health side? I think there have been some assessments of that and if I recall correctly*, *quite sizable chunks or proportions*, *and ratios of the COVID-19 funding went into some of the social*, *the economic elements of COVID-19.* (Respondent 15)

Moreover, many study respondents perceived an increased intersectoral and interdisciplinary collaborative work and improved communication flows among researchers, in both global and national public health. From their accounts, it seemed that these collaborations were not routinely fostered. They further expressed that these practices should be maintained in the long run.

*The only positive effect that I identified was the requirement for collaboration. Most of these projects involved collaboration between countries in the North (…). So the fact that the call [of IDRC WOMEN-RISE] required that there be this collaboration in my opinion helped to strengthen the work between the North and the South.* (Respondent 34)

*It [COVID-19] has forced and enabled much more engagement between social science perspectives*, *epidemiological and clinical perspectives*, *public health perspectives*, *and mathematical modelling*, *and so I think that’s really interesting and important. It has highlighted platforms*, *information flows*, *and communications in ways that could have long-term positive consequences.* (Respondent 35)

#### Unintended consequences related to information and data flow

Two of the most salient unintended consequences were the massive information flows stemming from COVID-19-related research in published journals and misinformation in social media. The volume of preprints multiplied, manuscript review time decreased drastically, and evidence-based decision-making turnaround was unprecedented. However, the evidence-based policy change was not necessarily observed nor were issues of (in)equities being addressed despite the availability of more data collected and knowledge shared.

*One [example] is just around the sheer pace of publication and the volume of publication. Preprints have absolutely exploded during COVID-19 and we have seen that when preprints are also accompanied by widespread press and social media attention that there can be some unintended consequences before the papers have gone through thorough peer review. (Respondent 18)*

*How are we going to manage this [social media]? I feel we should have government agents working actively on this*, *responding to the misinformation that we see online (…). What’s our communication strategy in the new age?* (Respondent 6)

Complementary to how knowledge and evidence were being used and disseminated was the increased attention on data measurement, availability, interoperability, and monitoring, in particular in the context of the Canadian federal system of several provincial and territorial healthcare and public health systems.

*There is a clear relationship*, *for example*, *the most deprived neighbourhoods have higher incidences of COVID-19 but all of these types of analyses*, *they require you to be adept at handling data. You have to be able to take Statistics Canada’s deprivation indices by postal code. You have to link that to our laboratory-confirmed COVID-19 cases and then you have to link that to immunisation registries (…). Those are the things that fall off the desk looking at an equity lens to COVID-19 because everyone is just like*, *we need to know the numbers today.* (Respondent 22)

#### Lessons learned expressed by study respondents

In the last two decades, different populations faced SARS-CoV-1, MERS, H1N1, Avian Flu, and Ebola outbreaks affecting thousands of people across regions of the world. Although COVID-19 is relatively recent in global public health history, three major lessons based on respondents’ input can be drawn based on past pandemics and current and past research on infectious diseases. The first one concerns collaborative work on emergency and pandemic preparedness coupled with the importance of the One Health approach where the interface between human, animal, and environmental health becomes key in addressing this current and future pandemics of zoonotic nature.

*Actually before the [COVID-19] pandemic*, *there were several international groups working on pandemic preparedness (…). Just in the past hundred years*, *we have got four influenza pandemics and we have two pandemics caused by coronaviruses. So I think that the direction would be to look at the pandemic threats of these respiratory virus infections and of course to look at something that we call the One Health approach when we are thinking about the pandemic threat because over 75% of these emerging infectious diseases are coming from an animal source*, *right? So*, *the pandemic is kind of assisted by all the other vectors like globalisation*, *the food industry*, *the proximity between humans and animals*, *and international travel and trade. We are kind of living in a global village now.* (Respondent 2)

The second lesson learned complements the more holistic examination of global and public health issues, focusing on multilevel systems and upstream determinants of health such as research funding and policy governance that might profoundly shape the health of populations downstream.

*[Regarding] the research agendas*, *more opportunities and funding became available but I don’t know if they were always asking the right questions. I think there has been some sort of*, *not as much emphasis on how the pandemic illuminates the systems that were already failing rather than suggesting that we can have Band-Aid quick-fix measures. I think it could have had more focus on*, *again*, *the need for widespread population-level interventions very far upstream on structural causes.* (Respondent 14)

As seen throughout the study respondents’ accounts, equity weaves through the different themes discussed in GHG, public health measures, population health research, COVID-19-related consequences, and lessons learned. Without adequate health equity impact assessments, we are globally subjected to repeating past failures and reproducing not only vaccine inequities but also systemic injustices as observed in this pandemic.

*The top three lessons of the pandemic I like to say are equity*, *equity*, *and equity (…). These are large numbers of people that are dying needlessly due to simply distributional effects related to vaccines. And of course*, *the long-term issue is not distributing vaccine commodities*, *it’s the local production so countries can be sustainable in their own vaccine production. Because as you get new waves and you get new pandemics*, *you are just going to get vaccine inequity 2.0. You are going to start up over and over again.* (Respondent 13)

### Recommendations of study participants to address inequities in COVID-19-related global health governance and population health research in Canada

To redress the numerous challenges and inequities raised, study respondents discussed several multipronged solutions and recommendations that we have regrouped below into three main categories. The recommendations answer ‘What, ‘How’, and ‘For Whom’ questions.

#### ‘What’ recommendations

Study participants mentioned the combination of adequate funding and allocation processes by different research agencies as one of the important upstream determinants for the realisation of equity-focused population health priorities, including research, and accountability outcomes as part of good governance, without which well-intentioned global health and population health objectives would be meaningless. It was further recommended that WHO as one of the central GHG actors should further receive adequate funding given its key role in the global public health arena.

*We can potentially think about how a solution moving forward is to continue to help to change the pendulum in terms of where the investments are going*, *where the energy is going in our research agenda more broadly but particularly our equity-focused research agenda.* (Respondent 31)

*[There is a] need to fix and fund properly WHO*, *which is underfunded. There is a lot of expertise and mechanisms for inclusive and diverse participation.* (Workshop group exercise participants)

More intersectoral collaboration and inter-regional coordination among interdisciplinary actors were suggested for policy decisions to be based on evidence and to deal with multilevel global health and populational health research issues, especially when there is an increased demand for the selection of indicators and data measurement and sharing to address COVID-19.

*The data wasn’t sufficient. We found so many cracks and holes in our data infrastructure whether it was just data that didn’t exist*, *you know that we just didn’t have or whether it was the inability to share data or whether it was the inability to measure things the same way*, *you know*, *in terms of interoperability or coordination of indicators.* (Respondent 20)

*The solution is greater cooperation*, *lifting all boats*, *making sure that capacities around the world*, *particularly in regions where these threats are more likely to emerge strengthening capacities for detection and response*, *strengthening health systems in general*, *and strengthening agreements internationally in how cooperation will occur so that when faced with something like this*, *even at a regional scale*, *there is a better capacity to respond.* (Respondent 10)

Beyond the traditional involvement of governmental entities, the participation of civil society, “community members” (Workshop group exercise members), and population groups in conditions of vulnerability or marginalisation was underscored. According to study respondents, they should be mobilised in population health research that concerns them and where their voices matter for long-term more sustainable outcomes.

*You can think about Indigenous populations in Canada*, *are we thinking about how to use data from Indigenous populations in a way that’s fair and respects their data governance principles even (…) If you did research that actively and meaningfully involved different communities*, *then we have more buy-in*, *into the products of that research*, *whether it’s a vaccine*, *whether it’s a therapy*, *whether it’s a diagnostic because you’ve participated in it.* (Respondent 5)

#### ‘How’ recommendations

Policy-related recommendations were also suggested by some study respondents to influence the population health research processes by explicitly addressing equity in the Canadian Institutes of Health Research (CIHR) Act beyond improving population health, which is not currently the case [48].

*The first thing we need to do is to change the basic law of CIHR and bring that back with inequality. Because the biggest funder of research in Canada is CIHR. So to bring that back into the concerns of the organisation. That is*, *not only must health research in Canada contribute to improving the health of Canadians*, *but it must also contribute to reducing health inequalities among Canadians.* (Respondent 33)

Another key recommendation on how global and public health research can structurally integrate considerations of equity stems from decisions and processes taken at the Canadian Cabinet-level where standards and procedures are set for subsequent equity outcomes to occur in other sub-levels and sectors.

*One of the ways to do that is to put in place standards and procedures to foreground equity in all aspects on the evidence-demand side. So*, *with Privy Council Office requiring their Memoranda to Cabinet which is what you need to do*, *the document you bring forward when you are seeking a cabinet decision*, *those documents should be required to present evidence that foregrounds an equity perspective and says*, *here is how the problem differentially affects different groups (…) So*, *you can have standards and procedures for Memorandum to Cabinet for Treasury Board submissions for so many things that happen in decision-making processes that just make it the new normal.* (Respondent 24)

The above recommendations were envisioned to be accompanied by accountability processes that can assess downstream which population groups have been particularly affected or who have not been considered in the design of research and intervention initiatives.

*If there are no actionable outcomes*, *should they be in the same position to do that next year? What hat are the measurable changes that they are making? Is there a decrease in particular health disparities? (…). There is no Black. There is no Asian. There are no Indigenous folks in this place. The health outcomes are in the water in this community (…). If there were no changes*, *then we have got to look into who is governing and that the governance is not working. And we have to make the changes.* (Respondent 27)

#### ‘For whom’ recommendations

Having discussed ‘What’ and ‘How’ recommendations, specific groups of actors in global public health were targeted to link recommendations with one another. The first group of actors is universities and public health schools, in addition to “data and social scientists and medical schools” (Workshop group exercise participants) that will train future generations of global and public health leaders, policy-makers, researchers, and practitioners to think of (in)equity and its impacts.

*To show that the doctoral schools [public health schools providing doctoral programmes] and the universities that train [researchers and practitioners] must also take a certain leadership role in this area [equity considerations]. Things are going fairly well in Canada*, *but in the rest of the world*, *in any case in the French-speaking world that I know*, *we are still very far from it. So there is certainly a need for significant change at that level. But once again*, *changing the training courses and those who go through the training courses is not enough as long as we don’t change the structures.* (Respondent 12)

Given the determinant role of research funding schemes, objectives, and priorities in shaping what research projects are being funded, actors involved in the design of funding grants and funding review panels were identified so they can be trained to shape what equity-focused research initiatives should encompass for actionable equitable health outcomes to materialise.

*Peer review policies or peer review standards*, *the cultural attitudes*, *and the attitudes of peer reviewers. All of these things in order for equity to be advanced*, *all of them need to be asking equity*, *we need to ask equity questions across all of them. And we have to thread those*, *we have to be able to look at the micro and the macro all at one time. We have to be able to move our gaze from close*, *very nearsighted to farsighted and back and forth.* (Respondent 14)

Canada’s national policy- and decision-makers were considered by study respondents to play an important role in fostering and pursuing multilateralism amid COVID-19-related GHG that would influence the outlook of public and population health research and interventions through an equity lens.

*There’s a real opportunity for Canada to translate its support of multilateralism into concrete action and that is through the WHO relationship. WHO is at the heart of the multilateral system. And so if Canada were*, *for example*, *to learn lessons from Germany*, *but to be a stronger multilateralist in word and deed in the important fora in which Canada participates*, *the G7*, *the G20*, *the Commonwealth*, *the Francophonie*, *and I think the partnership with WHO which is at the centre of the multilateral system in health is a good way to commit with that. And by the way*, *some of those fundamental issues going from equity to solidarity to empathy are not unique to the pandemic.* (Respondent 13)

## Discussion

This paper explored the multilevel interplay between GHG and Canadian population health research priorities in the context of COVID-19. Adopting a theoretical bricolage combining the conceptual strengths of the Intersectionality and Multiple Streams frameworks [39], this qualitative study enabled us to better understand the complex intersections among governance, global health, national public health, research priorities, and how the health of population groups through an intersectional lens remains at the centre of research foci. We report three main findings.

First, our study findings showed the stickiness of social and structural health inequities at the intersection of COVID-19-related GHG and population health research in Canada. Equity is a central tenet of both global and public health [49] and is also at the heart of GHG [50] and public health governance in Canada [51]. Although equity considerations were deemed important by the majority of study respondents, COVID-19-related inequities and systemic injustices remain unabated affecting the most vulnerable and marginalised within and across countries [2, 52, 53]. A recent scoping review which examined GHG and health equity in the context of COVID-19 found similar themes as addressed by our study respondents when expressing their recommendations on ‘What’ and ‘How’ to address COVID-19-related health inequities. This scoping review reported the importance and recurrence of seven main themes that emerged from the studies included in the review: (1) human rights and inequities (e.g., inequitable access to COVAX for COVID-19 vaccine), (2) the importance of solidarity, collaboration, and partnernship, (3) GHG structure change (e.g., related to structural factors for health inequity and adequate financing), (4) political and economic power and finance (e.g., unequal power relations in global health), (5) approaches to address inequity (e.g., mutual collective accountability), (6) law and regulations (e.g., the role of law), and (7) private investments and public-private partnerhships in GHG (i.e., that can cause inequity) [50]. As our findings suggest, our social contract for global and domestic solidarity has been breached [50]. In Canada, although the federal government seemed to have been an early responder to addressing COVID-19 domestically [54], the exacerbation of existing social inequities prevailed among elderly people, Indigenous communities, BIPOC, women and children in situations of abuse, people with disabilities, and people with substance use disorders [52]. The arsenal of public health measures to respond to COVID-19 such as lockdowns, social distancing, vaccination, and the use of applications for contact tracing and vaccination status acted as powerful tools to control both the virus and people’s health [55]. However, in the initial COVID-19 responses, explicit equity considerations were often relinquished to the background [5] although a collective call for solidarity was made to stay home to protect the health of vulnerable people and the capacity of the healthcare system to treat sick people [56]. In a time of global health crisis, it has been further suggested that the relational approach to ethics must focus on “solidarity, interconnectedness, transparency, and trust” if we aspire to a sustainable exit from COVID-19 and other future health crises such as climate change [56].

Second, discussing equity [50, 52, 57], funding [53], and accountability [58] in GHG and population health research separately or partly together [59] is not new. Rather, it exemplifies the dominantly siloed approach adopted in both global and public health governance to date. More than a decade ago, the issue of funding linked to priority setting and the need for accountability as a key domain of governance [60] in meeting global health goals was discussed given the multitude of global health actors and coordination challenges [59]. The Chief Public Health Officer of Canada reiterated in her 2022 annual report the priority to address COVID-19-related intersectional inequities accompanied by sustainable funding of public health [53]. However, what is new is the urgent recentring of the triad of funding (re-investment), equity (of all types), and accountability (responsibility and answerability [60]) in a common and integrated thread for better governance in both global health and population health research in Canada. It has been argued that unless power asymmetries are addressed and seriously acted upon between the Global North and Global South, donors and recipients including researchers, and mainstream groups and populations living and working in conditions of vulnerability or marginalisation, no equitable and accountable health outcomes are possible for various population sub-groups worldwide [61, 62]. Decolonising approaches to “use our privileges responsibly” is considered a necessary prerequisite to addressing the root causes of power asymmetries in global and public health governance by redistributing resources (technical, financial, and material) [53, 61]. Recently, Ted Schrecker, a Canadian political scientist, posed the provoking question of whether we are not collectively “building back worse” in the post-pandemic world with socioeconomic and health inequities that persist [63]. He argues that beyond rights, redistribution, and regulation [64], reinvestment is of utmost importance to reduce health inequities more sustainably [63]. Literature reports that different accountabilities from governmental agencies, public-private partnerships, and civil society actors will have to operate jointly and at different levels of governance for policy integration of desired changes and implementation of equitable initiatives including research to emerge [65].

Third, the COVID-19 pandemic has underscored the importance of considering the numerous lessons learned from past pandemics, and the specific contexts within which they occurred [66]. One of the main lessons learned was widespread pandemic unpreparedness in responding to COVID-19 globally and at the country level, including in Canada which scored one of the highest Global Health Security indexes among the Organisation for Economic Cooperation and Development (OECD) countries [67]. It has been argued that the cumulative failed COVID-19 preparedness processes and responses might have been influenced by concomitant tensions between health and the economy, international cooperation and local responses, and global solidarity and the local needs of citizens [68]. The availability of more robust data-sharing mechanisms that include interoperability of COVID-19 case reporting and data accessibility within and across countries might have improved how different national and sub-national jurisdictions and global actors such as WHO have responded to COVID-19 challenges [69]. Not only did the COVID-19 pandemic highlight the need for data availability, management, and analysis in real time, but it has also underscored the importance of transparent and coherent information flows among multilevel actors and in social media to counter misinformation and promote effective risk communication for public trust [53, 70]. Moreover, given the presumed zoonotic nature of COVID-19, adopting a One Health approach becomes crucial beyond getting ready for the next pandemic [71–73]. Additionally, a robust multisectoral approach combined with training of an interdisciplinary workforce is necessary while considering and acting upon structural and social determinants of health that shape shared human, animal, and environmental ecosystems [74].

### Limitations

This study has three main limitations. First, the perceptions of study participants were collected between February and August 2022, during the initial phases of the COVID-19 pandemic. Hence, they might have changed given the temporality of COVID-19 and the participants’ specific context (geographic location, population characteristics, and public health policy and measures put in place). However, as of November 2024, although most countries have removed the COVID-19-related public health measures, about 300,000 cases of COVID-19 continue to affect population health and health systems as reported by WHO [75]. Second, we did not interview political scientists or politicians and only 14% (5/35) of study respondents were French-speaking; they could have provided other perspectives on our research questions that have not been addressed. However, we interviewed study respondents who were cognizant of the various policy changes in their jurisdiction to respond to COVID-19. Third, less than 50% of WHO/international actors who were invited accepted to participate in the study. Among them, they mentioned their interest and the relevance of this timely study, however, could not help citing a busy schedule. Among those who participated, most were from the Global North which might have left the perspectives of WHO/international actors from the Global South.

## Conclusion

The current COVID-19 pandemic is unprecedented in its magnitude and impact. Study findings showed that there is still a unique collective opportunity to address the socioeconomic and health inequities caused by COVID-19 by taking into account the numerous recommendations made by the four different groups of study respondents. This study provides timely and substantial evidence of the multilayered and complex intersection between COVID-19-related GHG and population health research priorities in Canada through intersectional and multiple streams lens. Study findings illuminate the evolving COVID-19-related GHG architecture in terms of financial stewardship, accountability, and equity, and the consequences on health equity, population health, and research, including researchers, and the lessons to be learned. Furthermore, they will directly contribute to improving knowledge of global and national population health and equity research strategies in the context of an evolving pandemic. Global and public health policy- and decision-makers have the evidence to adjust and rectify the course of global public health. It is now our collective choice whether we are serious about addressing equity considerations in GHG and population health research in Canada and beyond.

## Electronic supplementary material

Below is the link to the electronic supplementary material.

Supplementary Material 1

## Data Availability

The datasets generated and/or analysed during the current study are not publicly available due to participant privacy and confidentiality considerations but are available from the corresponding author on reasonable request.

## Abbreviations

BIPOC: Black, Indigenous, and People of Colour
CIHR: Canadian Institutes of Health Research
GHG: Global Health Governance
IBPA: Intersectionality-Based Policy Analysis
IDRC: International Development Research Centre
MSF: Multiple Streams Framework
OECD: Organisation for Economic Cooperation and Development
SDG: Sustainable Development Goal
TRIPS: Trade-Related Aspects of Intellectual Property Rights
WHO: World Health Organization

## References

[1] WHO. WHO Coronavirus (COVID-19) Dashboard. Geneva: WHO. 2022 [cited on 14 Dec 2023]. Available: https://covid19.who.int/

[2] Sachs JD, Karim SSA, Aknin L, Allen J, Brosbøl K, Colombo F, et al. The Lancet Commission on lessons for the future from the COVID-19 pandemic. Lancet. 2022;400(10359):1224–80. 36115368 10.1016/S0140-6736(22)01585-9 PMC9539542

[3] Lee K, Kamradt-Scott A. The multiple meanings of global health governance: a call for conceptual clarity. Globalization Health. 2014;10(1):28. 10.1186/1744-8603-10-28 24775919 10.1186/1744-8603-10-28 PMC4036464

[4] Sarkodie SA, Owusu PAJE, Development. Sustainability. Global assessment of environment, health and economic impact of the novel coronavirus (COVID-19). Environ Dev Sustain. 2021;23(4):5005–15. 32837273 10.1007/s10668-020-00801-2 PMC7272320

[5] Mac-Seing M, Gidey M, Di Ruggiero E. COVID-19-related global health governance and population health priorities for health equity in G20 countries: a scoping review. Int J Equity Health. 2023;22(1):232. 37924074 10.1186/s12939-023-02045-8 PMC10625304

[6] Alcázar L, Bhattacharya D, Charvet E, Kida T, Mushi D, Ordóñez A, et al. COVID-19 in the Global South: impacts and policy responses. Occasional Paper Ser. 2021;69:1–49.

[7] Bowleg L. We’re not all in this together: on COVID-19, Intersectionality, and Structural Inequality. Am J Public Health. 2020;110(7):917. 10.2105/AJPH.2020.305766 32463703 10.2105/AJPH.2020.305766 PMC7287552

[8] Mac-Seing M, Rocha de Oliveira R. Health inequities and technological solutions during the first waves of the COVID-19 pandemic in high-income countries. Global Health Promotion. 2021. 10.1177/1757975920984185 33590801 10.1177/1757975920984185

[9] Danet AD. Psychological impact of COVID-19 pandemic in western frontline healthcare professionals. A systematic review. Medicina Clínica. 2021;156(9):449–58. 10.1016/j.medcle.2020.11.003 PMC7972644 33758782

[10] Sritharan J, Jegathesan T, Vimaleswaran D, Sritharan A. Mental health concerns of frontline workers during the COVID-19 pandemic: a scoping review. Global J Health Sci. 2020;12(11):89–105.

[11] The Lancet. The plight of essential workers during the COVID-19 pandemic. Lancet. 2020;395(10237):1587. 10.1016/S0140-6736(20)31200-9 32446399 10.1016/S0140-6736(20)31200-9 PMC7241973

[12] Oliveira MR, Sudati IP, Konzen VDM, de Campos AC, Wibelinger LM, Correa C, et al. Covid-19 and the impact on the physical activity level of elderly people: a systematic review. Exp Gerontol. 2021;159(111675):1–10. 10.1016/j.exger.2021.111675 PMC8695515 34954282

[13] Daoust J-F. Elderly people and responses to COVID-19 in 27 countries. PLoS ONE. 2020;15(7):e0235590. 32614889 10.1371/journal.pone.0235590 PMC7332014

[14] Hsu AT, Lane N, Sinha SK, Dunning J, Dhuper M, Kahiel Z et al. Impact of COVID-19 on residents of Canada’s long-term care homes–ongoing challenges and policy response. Canada: LTC Responses to COVID-19, International Long-Term Care Policy Network; 2020 [cited on 30 May 2020]. Available: https://ltccovid.org/2020/04/15/impact-of-covid-19-on-residents-of-canadas-long-term-care-homes-ongoing-challenges-and-policy-response/

[15] UNWOMEN. Paying attention to women’s needs and leadership will strengthen COVID-19 response. New York: UNWOMEN. 2020 [cited on 22 March 2020]. Available: https://www.unwomen.org/en/news/stories/2020/3/news-womens-needs-and-leadership-in-covid-19-response

[16] Nigam S. COVID-19, Lockdown and Violence against women in Homes. SSRN. 2020:1–10.

[17] Alon T, Doepke M, Olmstead-Rumsey J, Tertilt M. The impact of COVID-19 on gender equality. National Bureau of economic research; 2020.

[18] Wong JY-H, Wai AK-C, Wang MP, Lee JJ, Li M, Kwok JY-Y, et al. Impact of COVID-19 on child maltreatment: income instability and parenting issues. Int J Environ Res Public Health. 2021;18(4):1501. 33562467 10.3390/ijerph18041501 PMC7915078

[19] Ashikkali L, Carroll W, Johnson C. The indirect impact of COVID-19 on child health. Paediatrics Child Health. 2020;30(12):430–7. 32959000 10.1016/j.paed.2020.09.004 PMC7494255

[20] Marcelin JR, Swartz TH, Bernice F, Berthaud V, Christian R, da Costa C, et al. Addressing and Inspiring Vaccine confidence in Black, Indigenous, and people of Color (BIPOC) during the COVID-19 pandemic. Open Forum Infect Dis. 2021;8(9):ofab417. 10.1093/ofid/ofab417 34580644 10.1093/ofid/ofab417 PMC8385873

[21] Lopez PJ, Neely AH. Fundamentally uncaring: the differential multi-scalar impacts of COVID-19 in the US. Soc Sci Med. 2021;272:113707. 33517126 10.1016/j.socscimed.2021.113707 PMC8724555

[22] Power T, Wilson D, Best O, Brockie T, Bearskin LB, Millender E, et al. COVID-19 and Indigenous peoples: an imperative for action. J Clin Nurs. 2020;29(15–16):2737–41. 32412150 10.1111/jocn.15320 PMC7272911

[23] Turk MA, McDermott S. The COVID-19 pandemic and people with disability. Disabil Health J. 2020;13(3):100944. 32475803 10.1016/j.dhjo.2020.100944 PMC7254018

[24] Lebrasseur A, Fortin-Bédard N, Lettre J, Bussières E-L, Best K, Boucher N, et al. Impact of COVID-19 on people with physical disabilities: a rapid review. Disabil Health J. 2021;14(1):101014. 33158795 10.1016/j.dhjo.2020.101014 PMC7603994

[25] Perri M, Dosani N, Hwang SW. COVID-19 and people experiencing homelessness: challenges and mitigation strategies. Can Med Assoc J. 2020;192(26):E716–9. 10.1503/cmaj.200834 32601252 10.1503/cmaj.200834 PMC7828890

[26] Tsai J, Wilson M. COVID-19: a potential public health problem for homeless populations. Lancet Public Health. 2020;5(4):e186–7. 10.1016/S2468-2667(20)30053-0 32171054 10.1016/S2468-2667(20)30053-0 PMC7104053

[27] Kluge HHP, Jakab Z, Bartovic J, D’Anna V, Severoni S. Refugee and migrant health in the COVID-19 response. Lancet. 2020;395(10232):1237–9. 10.1016/S0140-6736(20)30791-1 32243777 10.1016/S0140-6736(20)30791-1 PMC7270965

[28] Spiritus-Beerden E, Verelst A, Devlieger I, Langer Primdahl N, Botelho Guedes F, Chiarenza A, et al. Mental health of refugees and migrants during the COVID-19 pandemic: the role of experienced discrimination and daily stressors. Int J Environ Res Public Health. 2021;18(12):6354. 34208243 10.3390/ijerph18126354 PMC8296172

[29] Pai M. Covidization of research: what are the risks? Nat Med. 2020;26(8):1159. 10.1038/s41591-020-1015-0 32719486 10.1038/s41591-020-1015-0

[30] Bramstedt K. The carnage of substandard research during the COVID-19 pandemic: a call for quality. J Med Ethics. 2020;46(12):803–7. 33004545 10.1136/medethics-2020-106494

[31] Strong M. Cancelling the Spring 2020 Project Grant competition. Canada: CIHR; 2020 [cited on 6 Dec 2022]. Available: https://cihr-irsc.gc.ca/e/51927.html

[32] CIHR. COVID-19 May 2020 Rapid Research Funding Opportunity Results. Canada: Government of Canada. 2020 [cited on 6 Dec 2022]. Available: https://cihr-irsc.gc.ca/e/52013.html

[33] Adam D. Scientists fear that ‘covidization’ is distorting research. Nature. 2020;588(7838):381–3. 33268880 10.1038/d41586-020-03388-w

[34] CIHR Institute of Population and Public Health. Strategic plan 2022–2026. Transforming public health. Ottawa: CIHR. 2022 [cited on 6 Dec 2022]. Available: https://cihr-irsc.gc.ca/e/documents/ipph_strategic-plan-20220613-en.pdf

[35] Azim T, Bhushan A, Del Rio Vilas VJ, Srivastava R, Wijesinghe PR, Ofrin R, et al. Public health research priorities for WHO on COVID-19 in the South-East Asia Region: results of a prioritization survey. Health Res Policy Syst. 2022;20(1):1–9. 36064411 10.1186/s12961-022-00862-x PMC9443619

[36] Polašek O, Wazny K, Adeloye D, Song P, Chan KY, Bojude DA et al. Research priorities to reduce the impact of COVID-19 in low-and middle-income countries. J Glob Health. 2022;12. 10.7189/jogh.12.09003 PMC9010705 35475006

[37] Oyebode O, Ramsay SE, Brayne C. Public health research in the UK to understand and mitigate the impact of COVID-19 and COVID-19 response measures. J Epidemioly Community Health. 2021;75(3):209–12. 10.1136/jech-2020-214997 33028615

[38] WHO. Health equity, Geneva. WHO; 2024 [cited on 4 Nov 2024]. Available: https://www.who.int/health-topics/health-equity#tab=tab_1

[39] Mac-Seing M. Legislation, health policy and the utilisation of sexual and reproductive health services by people with disabilities: a mixed methods study in post-conflict Northern Uganda. Montréal: Université de Montréal; 2021.

[40] Hankivsky O, Grace D, Hunting G, Giesbrecht M, Fridkin A, Rudrum S, et al. An intersectionality-based policy analysis framework: critical reflections on a methodology for advancing equity. Int J Equity Health. 2014;13(1):119. 25492385 10.1186/s12939-014-0119-x PMC4271465

[41] Crenshaw KW. Mapping the margins: intersectionality, identity politics, and violence against women of color. Stanford Law Rev. 1991;43:1241–99.

[42] Kingdon JW. A model of agenda-setting, with applications. Law Rev Mich State Univ Detroit Coll Law. 2001:331.

[43] Amri MM, Logan D. Policy responses to COVID-19 present a window of opportunity for a paradigm shift in global health policy: an application of the multiple streams Framework as a heuristic. Glob Public Health. 2021;16(8–9):1187–97. 10.1080/17441692.2021.1925942 34044747 10.1080/17441692.2021.1925942

[44] Marshall C, Rossman GB. Designing qualitative research: Sage; 2016.

[45] Mac-Seing M, Di Ruggiero E, Medhora R, Upshur R. The intersections of global health governance and population health research priorities in Canada: What has COVID-19 taught us?. Ottawa: Canadian Conference on Global Health; 2022 [cited on 1 Dec 2022]. Available: https://pheedloop.com/ccgh2022/site/sessions/?id=SES2I4KKL7DZT3Q0N

[46] Braun V, Clarke V. Using thematic analysis in psychology. Qualitative Res Psychol. 2006;3(2):77–101. 10.1191/1478088706qp063oa

[47] Fidler D. Architecture amidst anarchy: global health’s quest for governance. Artciles Maurer Fac. 2007;329.

[48] Government of Canada. Canadian Institutes of Health Research Act. Canada: Government of Canada; 2000 [cited on 23 Dec 2022]. Available: https://laws.justice.gc.ca/eng/acts/c-18.1/page-1.html

[49] Koplan JP, Bond TC, Merson MH, Reddy KS, Rodriguez MH, Sewankambo NK, et al. Towards a common definition of global health. Lancet. 2009;373(9679):1993–5. 19493564 10.1016/S0140-6736(09)60332-9 PMC9905260

[50] El Kheir-Mataria WA, El-Fawal H, Bhuiyan S, Chun S. Global Health Governance and Health Equity in the Context of COVID-19: a scoping review. Healthcare. 2022;10(3):540. 35327017 10.3390/healthcare10030540 PMC8949542

[51] Di Ruggiero E, Bhatia D, Umar I, Arpin E, Champagne C, Clavier C, et al. Governing for the public’s health: governance options for a strenghtened and renewed public health systems in Canada Report 2022. Montreal: National Collaborating Centres for Public Health; 2022.

[52] PHAC. From risk to resilience: An equity approach to COVID-19. The Chief Public Health Officer of Canada’s Report. Ottawa: PHAC. 2020 [cited on 4 Nov 2021]. Available: https://www.canada.ca/content/dam/phac-aspc/documents/corporate/publications/chief-public-health-officer-reports-state-public-health-canada/from-risk-resilience-equity-approach-covid-19/cpho-covid-report-fra.pdf

[53] PHAC. A vision to transform Canada’s public health system. The Chief Public Health Officer of Canada’s Report. Ottawa: Public Health Agency of Canada; 2021 [cited on 26 Dec 2022]. Available: https://www.canada.ca/content/dam/phac-aspc/documents/corporate/publications/chief-public-health-officer-reports-state-public-health-canada/state-public-health-canada-2021/cpho-report-eng.pdf

[54] Lee K, Akuffo E, Shaw TM. Canada’s Covid-19 response: navigating national and global solidarity. Round Table 2020;109(3):326–7.

[55] Constantinou CS. Responses to Covid-19 as a form of ‘biopower’. Int Rev Sociol. 2022;32(1):29–39.

[56] Jeffrey DI. Relational ethical approaches to the COVID-19 pandemic. J Med Ethics. 2020;46(8):495–8. 32522813 10.1136/medethics-2020-106264 PMC7316115

[57] Hassan I, Mukaigawara M, King L, Fernandes G, Sridhar D. Hindsight is 2020? Lessons in global health governance one year into the pandemic. Nat Med. 2021;27(3):396–400. 10.1038/s41591-021-01272-2 33723454 10.1038/s41591-021-01272-2

[58] Legge DG. COVID-19 response exposes deep flaws in global health governance. Global Social Policy. 2020;20(3):383–7.

[59] Gostin LO, Mok EA. Grand challenges in global health governance. Br Med Bull. 2009;90:7–18. 10.1093/bmb/ldp014 19376802 10.1093/bmb/ldp014

[60] Bovens M. Analysing and assessing accountability: a conceptual framework. Eur Law J. 2007;13(4):447–68.

[61] Abimbola S, Asthana S, Montenegro C, Guinto RR, Jumbam DT, Louskieter L, et al. Addressing power asymmetries in global health: imperatives in the wake of the COVID-19 pandemic. PLoS Med. 2021;18(4):e1003604. 33886540 10.1371/journal.pmed.1003604 PMC8101997

[62] Friel S, Townsend B, Fisher M, Harris P, Freeman T, Baum F. Power and the people’s health. Soc Sci Med. 2021;282:114173. 34192622 10.1016/j.socscimed.2021.114173

[63] Schrecker T. Building back worse? The Prognosis for Health Equity in the post-pandemic world. In: Dalingwater L, Boullet V, Costantini I, Gibbs P, editors. The unequal costs of Covid-19 on Well-being in Europe. Switzerland: Springer; 2022. pp. 21–39.

[64] Labonté R, Schrecker T. Rights, redistribution, and regulation. In: Labonté R, Schrecker T, Packer C, Runnels V, editors. Globalization and health: pathways, evidence and policy. Routledge; 2009. pp. 339–55.

[65] Karlsson-Vinkhuyzen S, Dahl AL, Persson Å. The emerging accountability regimes for the Sustainable Development Goals and policy integration: friend or foe? Environment Planning C: politics and space. 2018;36(8):1371–90.

[66] Jones DS. History in a Crisis—lessons for Covid-19. New England Journal of Medicine; 2020. 10.1056/NEJMp2004361 32163699

[67] Abbey EJ, Khalifa BAA, Oduwole MO, Ayeh SK, Nudotor RD, Salia EL, et al. The Global Health Security Index is not predictive of coronavirus pandemic responses among Organization for Economic Cooperation and Development countries. PLoS ONE. 2020;15(10):e0239398. 10.1371/journal.pone.0239398 33027257 10.1371/journal.pone.0239398 PMC7540886

[68] Dentico N. The breathing catastrophe: COVID-19 and Global Health Governance. Development. 2021;64(1):4–12. 10.1057/s41301-021-00296-y 34276166 10.1057/s41301-021-00296-y PMC8275911

[69] LoTempio J, Spencer D, Yarvitz R, Delot-Vilan A, Vilain E, Delot E. We can do Better: lessons learned on data sharing in COVID-19 pandemic can inform future outbreak preparedness and response. Sci Dipl. 2020;9(2).

[70] You J. Lessons from South Korea’s Covid-19 policy response. Am Rev Public Adm. 2020;50(6–7):801–8.

[71] Acharya KP, Subramanya SH, Neupane D. Emerging pandemics: lesson for one-health approach. Veterinary Med Sci. 2021;7(1):273. 10.1002/vms3.361 PMC7675301 33030806

[72] Leifels M, Khalilur Rahman O, Sam I, Cheng D, Chua FJD, Nainani D, et al. The one health perspective to improve environmental surveillance of zoonotic viruses: lessons from COVID-19 and outlook beyond. ISME Commun. 2022;2(1):1–9. 36338866 10.1038/s43705-022-00191-8 PMC9618154

[73] Ruckert A, Zinszer K, Zarowsky C, Labonté R, Carabin H. What role for one health in the COVID-19 pandemic? Can J Public Health. 2020;111(5):641–4. 32909226 10.17269/s41997-020-00409-z PMC7480204

[74] FAO, OIE, WHO. Taking a multisectoral, One Health approach: A tripartite guide to addressing zoonotive diseases in countries. Geneva:: WHO; 2019 [cited on 26 Dec 2022]. Available: https://www.who.int/initiatives/tripartite-zoonosis-guide

[75] WHO. Number of COVID-19 cases reported to WHO. Geneva:: WHO; 2024 [cited on 4 Nov 2024]. Available: https://data.who.int/dashboards/covid19/cases?n=c

