# Supplementary material for: The complexity of addressing equity in COVID-19-related global health governance and population health research priorities in Canada: a multilevel qualitative study
Source: BMC Public Health. 2024 Dec 5;24:3381. doi: 10.1186/s12889-024-20893-z (PMC11619409; doi:10.1186/s12889-024-20893-z)
Supplement: Supplementary file 1 — Supplementary Material 1 [file 12889_2024_20893_MOESM1_ESM.docx]

**Key informant semi-structured interview guide – via Zoom video conference**

**Title of research study:** Redefining population health research agendas in Canada: A multilevel analysis of COVID-19-related global health governance architecture

| **Sex:** | **Background of participants:**   - Canadian researcher - Research institutes/universities in Canada - Research funding organisations - WHO/international-related |
| --- | --- |
| **Province/Country:** |  |

**Introduction**

Thank you for accepting to talk to me today. The interview will take up to a maximum of 60 minutes of your time. The information that you will provide will be kept confidential. Your answers will not be associated with your name unless you want to be identified. Your answers will only be shared with other research team members and will be compiled with other answers from various study participants.

I am going to ask you questions on how the COVID-19 related global health governance is redefining population health research agendas in Canada. At any time, you may ask clarification if the questions are not clear to you.

**Confidentiality**

Although the interview will be recorded, I would like to assure you that our discussion will remain confidential. The recordings will be kept safely until they are transcribed word for word, then they will be destroyed. The transcribed notes of the interview will contain no information that would allow individual participants to be linked to specific statements. If there are any questions or discussions that you do not wish to answer or participate in, you do not have to do so.

**Warm-up**

- Tell us a bit about yourself, your role and where are you working, and for how long have you been with this organisation?

**Guiding questions**

The following two sets of questions compose the overall list of potential interview questions. During each interview, depending upon the interests and background of study participants, a sub-set of questions will be selected from this overall list of questions.

1. **Descriptive questions related to the intersectional and multiple streams frameworks**

For the following questions, at any time, you can also provide specific examples from your research or that of your organisation

- Based on your experience, how would you describe population health research in Canada?
- What can you tell about the population health research agendas in Canada, especially related to health equity and the links to social determinants of health?
- What do you know about the features (such as coordination and accountability mechanisms) of the global health governance architecture in Canada and how does it relate to the current COVID-19 pandemic?
  - Which (policy) actors are most involved in this COVID-19-related global health governance in Canada?
- In your opinion, how has the governance of Canadian population health research agendas been impacted by the COVID-19 pandemic? (Probe for research project objectives, scope, types of collaboration, and activities)
  - Are there any observed changes in terms of policies and research agendas at the Canadian level?
  - Any specific elements from the political side that might have contributed to these changes?
  - Any specific (policy) trigger (window of opportunity)?
- Reflecting on your experience since the onset of the COVID-19 pandemic, are there any consequences – unintended, positive, negative, or no changes – on [your or your institution] population health research initiatives and aspects such as health equity and social determinants health?
  - Any specific population groups have been more affected than the others, and why would you say so?
  - Can you comment on how health equity issues have been addressed (or not) in the COVID-19-related global health governance for population health research agendas in Canada?
  - Without the COVID-19 pandemic, do you think these changes/these situations would have occurred or not?

1. **Transformative questions related to the intersectional framework**

- In your opinion, how can we address the (mentioned) health inequities and unintended impacts on certain groups of marginalised and vulnerable populations through research, and other actions?
- What are some of your recommendations or solutions in regard to more equity-focused COVID-19-related global health governance and population health research agendas in Canada?
- In your opinion, how would you describe the relationships among researchers, research institutes, research funders, and WHO actors?
  - Are there any specific aspects that need to be addressed for recommendations/solutions to be implemented in the future? (Probe: power dynamics)
- In your capacity as [background], what can be your role in addressing these issues or be part of the process in solution-finding?
- Compared to other places in Canada, countries, or international GHG actors such as WHO, what can we learn from the COVID-19 global health governance and population health research in Canada?

**Conclusion**

- Thank you for participating. This has been a very successful discussion
- Your opinions will be a valuable asset to the study
- We hope you have found the discussion interesting
- I would like to remind you that any comments featuring in this report will be anonymous
